# Effect of Coronal Flaring on Initial Apical File Size Estimation in Curved Canals Using Three Distinct Rotary Instruments: A Comparative In Vitro Study

**DOI:** 10.7759/cureus.56687

**Published:** 2024-03-22

**Authors:** Vinodhini Varatharajan, Muhammed Abdul Rahman Thazhathveedan, Mohammed Salman Kuttikkodan, Ismail Puzhangaraillath Mundanatayil, Amrutha Ravindran Thazhe Mangool, Ashraf Karumbil

**Affiliations:** 1 Conservative Dentistry and Endodontics, Mahatma Gandhi Postgraduate Institute of Dental Sciences, Puducherry, IND; 2 Conservative Dentistry and Endodontics, Educare Institute of Dental Science, Malappuram, IND; 3 Dentistry, Dent Inn Multispeciality Dental Clinic, Kozhikode, IND; 4 Conservative Dentistry and Endodontics, Sree Anjaneya Institute of Dental Sciences, Kozhikode, IND

**Keywords:** preflaring, mesiobuccal root, initial apical file size, hyflex cm rotary niti files, endoflare rotary instrument

## Abstract

Background and objectives: The initial size of a root canal is established by progressively introducing K-files according to the increase in the International Organization for Standardization (ISO) size in the apical region. The initial file-fit sensation is caused by coronal interferences rather than always occurring at the apex, as is commonly believed. Flaring the canal at its earliest stages enables the practitioner to accurately assess the size of the canal approaching the apex. This enables more informed judgments on the selection of the master apical file required for shaping and cleaning the apex. The aim of this in vitro study is to examine the impact of cervical flaring on the first estimation of apical file size using three distinct rotary instruments.

Materials and methods: Sixty-four extracted permanent maxillary first molars with a curvature of between 10⁰ and 20⁰ were chosen. Conventional access openings were made, and the precise length of the canal was determined, leaving it 1 mm short of the apex. The apical fit was deemed to have materialized when the largest file successfully reached the apex, and further progression beyond that depth was unattainable. An initial file that exhibited tactile resistance both before and following expansion at the designated working length (WL) was observed. The initial file that elicited a sensation of being securely attached was affixed using methacrylate into the root canal. A diamond sectioning disc was used to horizontally cut the apical 0.5 mm of the mesiobuccal root. This was done to expose the canal and the instrument at the WL. The uppermost portions were observed using a 3D optical profilometer, and digital photographs were captured for each sample.

Results: The occurrence of coronal interferences and the choice of instruments for flaring had a notable impact on the estimation of the initial apical file (IAF) size. The file size frequency was augmented following flaring using various rotary instruments, namely ProTaper, HyFlex CM, and Endoflare. Group 1, which did not undergo preflaring, exhibited the highest disparity of 257.3 ± 54.4. The variation was substantially different (p<0.01) from all the groups that underwent flaring. The use of HyFlex CM (group 3) for preflaring resulted in the smallest average difference (124.4 ± 29.6) between the maximum diameter of the canal at the apex and the diameter of the initial file used. Endoflare (group 4) exhibited the second lowest mean disparity (178.7 ± 46) between the maximum width of the apical root canal and the diameter of the IAF, with the ProTaper group (211 ± 43.5) following closely behind. Nevertheless, there was no statistically significant discrepancy observed in the average differences between groups 2 and 4 (ProTaper and Endoflare groups, respectively).

Conclusion: Coronal preflaring significantly contributes to minimizing the variation between the IAF and the diameter of the apical canal. Prior coronal expansion using rotary files enables a more precise identification of the IAF. The choice of equipment used for flaring affects the estimation of the IAF size.

## Introduction

The two primary goals of endodontic therapy are cleaning and shaping the canal system, followed by 3D obturation [1]. During the process of cleaning and shaping the root canal, the clinician needs to assess three clinical factors: the canal's length, the preparation's taper, and its horizontal measurement at its most apical point, also known as the initial apical file (IAF) size [2]. More debris clearance efficiency is made possible by the increased canal volume, which also permits the administration of a maximum quantity of irrigation fluid and medication to the canal's most apical portions [3].

Efficient canal cleaning requires precise identification of the working length (WL) and sufficient widening of the apex of the canal. The degree of apical expansion is generally determined by approximating the initial dimensions of the canal apex. The conventional approach for determining the extent of the apical preparation involves sequentially advancing larger instruments to the WL until one encounters resistance, establishing the preoperative canal width. The apical file size calculation and tactile apical constriction detection rely on the presumption that the file freely enters the coronal and middle thirds of the canal and approaches the apical constriction [4]. Nevertheless, it has been shown that the perception of the IAF fit does not always result from contact at the apex, as commonly envisioned. Rather, it may be caused by an obstruction in the coronal and middle parts of the root canal. The unevenness of the walls and the curved shape of the root provide inaccurate pressure on the file and hinder the clinician's ability to assess contact and snugness at the apex [5].

The ongoing formation of secondary dentin gradually decreases the size of the pulp throughout an individual's entire life. There is an assertion that a daily deposition of 4 microns of secondary dentin occurs. The process of dentin formation is characterized by a slow and steady rate, which becomes more pronounced after individuals reach their middle decades of 35 to 40 years [6]. The highest restriction in molars is observed on the pulpal floor, while minimal constriction is noted on the side walls. Consequently, pulp chambers diminish dramatically in the occlusoradicular axis, whereas they do not decline in the mesiodistal axis when individuals are older [7]. To ensure precise measurement of the WL and IAF size, it is necessary to relieve this constriction through coronal flaring.

Research has shown that initiating flaring at an early stage is crucial for accurately determining the size of the apex through touch. The apical diameter was identified in merely 32.3% of the instances without early flaring; however, this percentage ascended to 75% after implementing this approach [8]. The size of the master apical file (MAF) has been correlated with the IAF in numerous investigations. The rule of increasing the size of the file by three sizes while binding is still being applied in several modified versions [9]. Research has indicated that conducting an initial flaring before calculating the apical size can provide a more precise estimation of the apex [2,10].

Gani and Visvisian documented the intricate forms found in the uppermost part of the root canals, specifically focusing on the first permanent molar in the upper jaw [11]. The apical portion predominantly exhibited circular patterns in the palatal roots, flat forms in the mesiobuccal roots, and a combination of circular and flat patterns in the distobuccal roots. The anatomical characteristics of the maxillary first permanent molar provide challenges for endodontic treatment, especially in the buccal canals. Curved canals can result in a deviation of the measuring instrument and elevate the level of resistance to friction [12]. Consequently, these curves have a distinct impact on a clinician's ability to feel through touch, resulting in a decrease in the precision of determining the initial WL. If there is a significant curve in the root canal, it is recommended to widen the upper part of the canal to make it easier to insert files into the lower part and to avoid putting too much pressure on the nickel titanium (NiTi) instruments [13].

Preparation for flare can be accomplished by employing either manual or rotary instruments. Previous studies have mentioned that it is beneficial to eliminate coronal third obstruction before doing apical instrumentation. Multiple studies have suggested that modifying the upper third of the canals affects the dimensions of the file that fits tightly at the tip [14,15]. Advancements in this domain have led to the creation of novel types of rotary tools that are specifically engineered for cervical flaring. The NiTi rotary system surpasses standard Gates Glidden drills in several aspects, including its exceptional flexibility, superior resilience to cycle fatigue, and enhanced cutting efficiency. The choice of instrument for coronal flaring is crucial in estimating the anatomical dimension at the WL [16].

A NiTi rotary instrument called the HyFlex CM (Coltène Whaledent, Switzerland) is made of controlled-memory wire, or CM-Wire, which enables the instrument to follow the anatomy of the canal and lowers the risk of ledging, transportation, and perforation. The cross-section of the HyFlex CM 25/0.04 is quadrangular, while that of the HyFlex CM 25/0.06 is triangular [13].

The ProTaper (Dentsply-Maillefer, Ballaigues, Switzerland) system features a progressive taper design with multiple shaping and finishing files, each serving a specific function in the root canal preparation process. The files have ProTaper, which has a convex triangular cross-section with a variable taper along the length of the instrument to effectively remove dentin while minimizing the risk of transportation and ledging. Files with increasing tapers from D1 to F2 facilitate efficient coronal, middle, and apical shaping of root canals. For an S-shaped design, files with a unique S-shaped cross-section enhance cutting efficiency and debris removal while reducing the risk of instrument binding [3].

The coronal portion of the root canal can be flared using EndoFlare (Micro-Mega, Besançon, France) prior to chemomechanical preparation. The file features a 10-mm blade length, a 0.12 taper, and a #25 tip size. EndoFlare files feature rounded tips to minimize the risk of perforation and damage to the root canal walls during coronal flaring [3].

Furthermore, despite the widespread use of a number of rotary file systems, including ProTaper, HyFlex CM, and EndoFlare, for coronal flaring, a limited comparative study has been done to assess each system's effectiveness in this setting. Hence, the study aimed to examine the impact of coronal flaring on the assessment of the IAF size in the mesiobuccal root of the maxillary molar with a curvature ranging from 10⁰ to 20⁰ utilizing ProTaper, HyFlex CM, and Endoflare rotary equipment.

## Materials and methods

The present in vitro study was conducted in the Department of Conservative Dentistry and Endodontics, Government Dental College, Calicut, collaborating with the Department of Mechanical Engineering at the National Institute of Technology in Calicut. The study was approved by the Institutional Ethics Committee (IEC No. 95/2016/DCC).

Sample selection and collection

The extracted maxillary first molar was collected, exhibiting three separate root canals, a fully formed apex, and a typical pulp chamber with a mesiobuccal root curvature between 10⁰ and 20⁰ as determined by Schneider's technique [17]. These molars had a single canal in the mesiobuccal root. The root surface of the teeth was examined using a stereomicroscope (x40) (Motic Smz-168 Series, Motic Asia in Hong Kong). Roots exhibiting fractures or external imperfections, open apices, intricate anatomic deviations with mild and severe curvature, calcified canals, external or internal resorption, long oval and irregular canal layouts, and those with root canal fillings and posts were excluded.

The sample size in each of the groups was estimated using the following calculation: n = (zα + zβ)2×SD2×2/d2, where n represents the sample size, SD is the standard deviation, Zα is 1.96 for α = 0.05, Zβ is 0.84, β is 0.20, and d represents the clinically relevant effect size. The standard deviation for variations (measured in microns) between the IAF diameter and the canals at the WL was found to be 0.002 in a previous study conducted by Sharma et al. [18]. Additionally, the discrepancy in means (d) was also set at 0.002. Consequently, the study required a minimum of 16 samples in every group.

Preparation of the sample

The teeth were thoroughly cleaned of soft tissues and calculus using a scaler, subsequently rinsed with running water, and finally preserved in a saline solution before the procedure. The teeth were affixed to wax for convenient manipulation. The cusps of the teeth were flattened to provide a flat occlusal surface, which allowed for a more precise estimation of the WL.

Preparation of the access cavity

The access cavities were created using round steel burs #4 and #6 (KG Sorensen, São Paulo, Brazil) and Endo-Z bur (Dentsply Maillefer, Ballaigues, Switzerland) at a high speed with the use of water spray. The pulp tissue was extracted using a barbed broach, ensuring no contact was made with the walls of the root canal. Subsequently, the canals received irrigation with a generous amount of a 3% sodium hypochlorite solution.

Canal sizing

To ensure the patency of the canal, a Dentsply International Organization for Standardization (ISO) 06/0.02 K-file was placed until it protruded from the apex. Following the maintenance of patency, an ISO 08/0.02 K-file was gently placed into the watch's winding movement until the file's tip was evident at the apex. The total length of the canal was found by inserting the k-file into the canal until it reached the apex. The WL was subsequently determined to be 1 mm shorter than this measurement. The apical fit was deemed to be achieved when the largest file approached the apex, and further advancement beyond that point was not feasible. The maximum file size that met the requirements and reached the specified length had been established for each group. In every case, a larger file was attempted to prevent it from reaching an identical WL. After selecting the biggest file, radiographs were captured from both the proximal and clinical perspectives. These radiographs confirm that the file has attained the appropriate WL and is well-fitted in the canal. The initial file fitting at the apex before flaring (FFFAb) was used for recording the file's size.

Following the estimation of the WL and starting apical file size, the teeth were categorized into four groups, with a total of 16 teeth in each group (N = 16). Coronal flaring was conducted in experimental groups 2, 3, and 4 with three distinct rotary devices. The process of rotary instrumentation was carried out using the Canal Pro-CL2 Endomotor, Coltene. The Canal Pro CL2 motors are wireless handpieces utilized in conjunction with rotary files to expand root canals. Various rotational speeds and torque values can be adjusted within the range of 120-600 rpm, with a maximum peak torque of 3.0 Ncm as shown in Table 1.

**Table 1:** Categorization of teeth into four groups IAF: initial apical file, PTU: ProTaper universal, CM NiTi: continuous molybdenum nickel titanium

Group	Description
Group 1 (control group)	The root canals were treated with the IAF technique but without any widening of the upper part of the tooth.
Group 2 (ProTaper group)	Utilized the SX ProTaper file from the PTU system by Dentsply Maillefer for preparing the root canals. Operated at a speed of 300 revolutions per minute with a torque of 2 Newton centimeters. Root canals regularly flushed with a 3% solution of sodium hypochlorite.
Group 3 (HyFlex CM Coltene group)	Utilized HyFlex CM NiTi files (08/25) for preparing the root canals. Operated at a speed of 500 revolutions per minute with a torque of 2.5 Newton centimeters. Root canals regularly flushed with a 3% solution of sodium hypochlorite.
Group 4 (Endoflare Micro-Mega group)	Utilized the Endoflare (12/25) instrument to prepare the root canals. Operated at a speed of 500 revolutions per minute with a torque of 2.5 Newton centimeters. Root canals regularly flushed with a 3% solution of sodium hypochlorite.

Establishing the IAF

**Figure 1:**
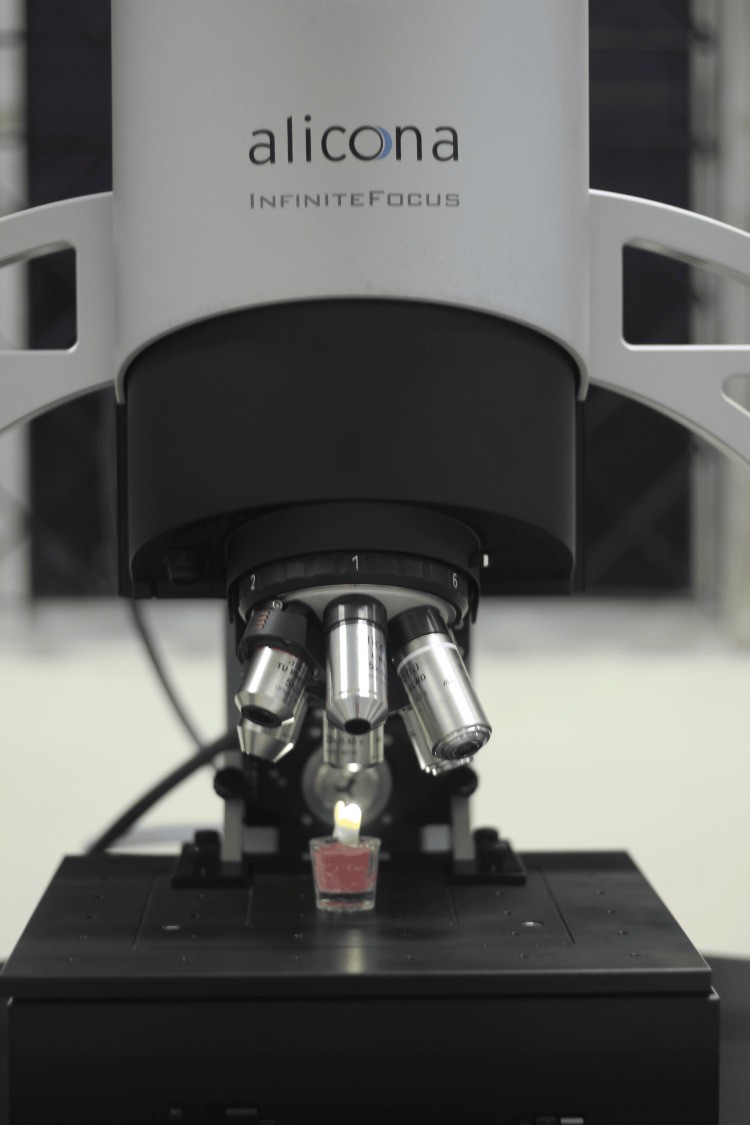
Setup of the microscope used for the analysis

**Figure 2:**
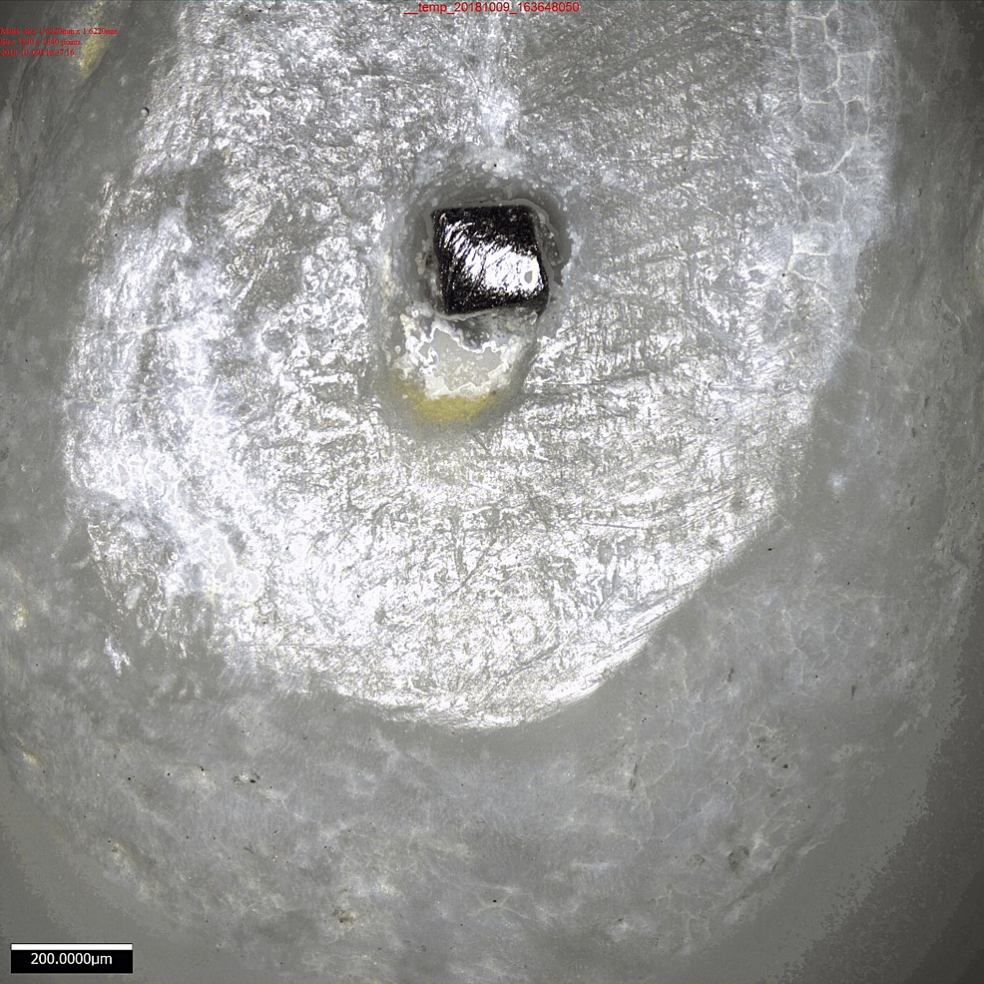
Surface image of the tooth analyzed

Following preflaring, manual files were introduced into the mesiobuccal root canal, beginning with a k-file ISO 08/0.02 at the established WL. The file size increased over time until a tiny amount of friction was detected at the WL. The initial file that exhibited a constricting feeling at the optimal WL was identified and then secured using self-curing acrylic resin within the root canal. The apical 0.5 mm of each root was horizontally eliminated employing a diamond sectioning disc, which was 0.2 mm thick and had a diameter of 1 inch (manufactured by Edenta, Switzerland). This procedure was done to expose the canal and the instrument at the WL. Subsequently, all teeth were subjected to a 0.5% sodium chloride solution and distilled water for 15 minutes each. The uppermost portions were observed employing a 3D optical profilometer (Alicona infinite focus G5) at a magnification of x10, and digital photographs were captured for each sample. The 3D optical profilometer employed in this study is a non-contact technique that visually scans the entire surface, producing a 3D depiction (Figures 1-2).

Analysis of images

The acquired images were analyzed using the integrated Insize MS software on a computer equipped with a 3D optical profilometer (Alicona infinite focus G5). It was employed to ascertain the dimensions of the canal and the IAF. The maximum width of the canal and that of the instrument were documented. The disparities among these variables were subjected to statistical evaluation and were subjected to a Wilcoxon-signed rank test to contrast the IAF that was fitted at the highest point before and after flare. The Kruskal-Wallis test was conducted to examine the incidence of file size escalation following flaring. The Mann-Whitney U test was conducted to determine the incidence of file size growth among several experimental groups. The data collected from this study underwent statistical analysis using SPSS Statistics version 25.0 (IBM Corp. Released 2017. IBM SPSS Statistics for Windows, Version 25.0. Armonk, NY: IBM Corp.). A one-way analysis of variance (ANOVA) was conducted to evaluate the differences observed between the maximum diameter of the binding instrument and that of the root canals. The mean value disparities between groups were compared using Scheffe's multiple comparisons test.

## Results

A Wilcoxon-signed rank test was conducted to contrast the initial file placement at the apex before and after flaring in three groups: group 2 (ProTaper group), group 3 (HyFlex CM group), and group 4 (Endoflare group) (Table 2).

**Table 2:** Comparison of FFFAb and FFFAa among the experimental groups FFFAb: apex before flaring, FFFAa: apex after flaring, SD: standard deviation p-value was considered significant if <0.05

Groups	FFFAb		FFFAa		p-value
	Mean ± SD	Median	Mean ± SD	Median	
Group 2	14.1 ± 2.7	15	21.9 ± 3.1	20	0.01
Group 3	14.1 ± 2.7	15	26.9 ± 2.5	25	0.01
Group 4	14.1 ± 2	15	25.9 ± 2	25	0.01

The p-value (<0.01) signifies a statistically significant difference between FFFAb and FFFAa within group 2 (ProTaper group), group 3 (HyFlex CM group), and group 4 (Endoflare group).

A Kruskal-Wallis test was used to examine the frequency of file size growth following flaring, which showed a significance level of p=0.01, indicating that there was a substantial variation in the upsurge of FFFA levels among group 2 (ProTaper group), group 3 (HyFlex CM group), and group 4 (Endoflare group), as shown in Table 3.

**Table 3:** Frequency of initial IAF increase among the experimental groups FFFAa: apex after flaring, SD: standard deviation, IAF: initial apical file p-value was considered significant if <0.05

Increase in FFFAa	Group 2		Group 3		Group 4		p-value
	Count	Percent	Count	Percent	Count	Percent	
5	7	43.8	0	0.0	0	0.0	0.01
10	9	56.3	7	43.8	10	62.5	
15	0	0.0	9	56.3	6	37.5	
Mean ± SD	7.8 ± 2.6		12.8 ± 2.6		11.9 ± 2.5		
Median	10		15		10		

The average frequency of file size increases was greater in group 3 (HyFlex CM group) and lesser in group 2 (ProTaper group). The Mann-Whitney U test was utilized to appraise the difference in frequency of file size increase after flaring between the groups, which indicates a statistically highly significant difference (p=0.01) between groups 2, 3, and 4, as shown in Table 4.

**Table 4:** Comparison in frequency of file size increase after flaring p-value was considered significant if <0.05

Comparing groups	Z-value	p-value
Group 2 vs. group 3	3.96	0.01
Group 2 vs. group 4	3.55	
Group 3 vs. group 4	1.05	

A one-way ANOVA was conducted to assess the significant differences in the disparity between the diameters of the instrument and canal across four groups. The statistical significance of the variance in the disagreement between instrument and canal diameters among the control group (group 1), ProTaper group (group 2), HyFlex CM group (group 3), and Endoflare group (group 4) is indicated by p=0.01. The average difference in diameter between the instrument and canal is greatest in group 3 (HyFlex CM group) and smallest in group 1 (control group) (Table 5).

**Table 5:** Comparison of discrepancy between diameters of instrument and canal among the experimental and control groups SD: standard deviation p-value was considered significant if <0.05

Group	Mean	SD	N	F-test	p-value
Group 1	257.3	54.4	16	25.46	0.01
Group 2	211.0	43.5	16		
Group 3	124.4	29.6	16		
Group 4	178.7	46.0	16		

Scheffe multiple comparisons were conducted to assess the differences in mean differences among groups, indicating a statistically highly significant difference in the diameters of the instrument and canal between the control group (group 1) and the HyFlex CM group (group 3), the control group (group 1) and the Endoflare group (group 4), and the ProTaper group (group 2) and the HyFlex CM group (group 3). There was a statistically significant difference in the diameters of the instrument and canal between group 1 (control group) and group 2 (ProTaper group) (p=0.042). Similarly, there was a statistically highly significant difference (p=0.011) in the diameters of the instrument and canal between group 3 (HyFlex CM group) and group 4 (Endoflare group). There was no statistically significant difference in the diameters of the instrument and canal between group 2 (ProTaper group) and group 4 (Endoflare group) (p=0.246), as shown in Table 6.

**Table 6:** Scheffe multiple comparisons to assess the differences in mean differences among groups p-value was considered significant if p<0.05

Scheffe’s multiple comparisons		
Comparison pair	F-test	p-value
1 & 2	2.91	0.042
1 & 3	24	0.01
1 & 4	8.4	0.01
2 & 3	10.19	0.01
2 & 4	1.42	0.246
3 & 4	4.01	0.011

## Discussion

The present investigation aimed to determine the impact of coronal flaring on the estimation of IAF size by employing three distinct rotating instruments (ProTaper, HyFlex CM, and Endoflare), utilizing 64 extracted permanent maxillary first molars. The occurrence of coronal interferences and the choice of instruments for flaring had a notable impact on the estimation of the IAF size. The file size frequency was augmented following flaring using various rotary instruments, including ProTaper, HyFlex CM, and Endoflare. When opposed to root canals without flaring, the HyFlex CM and Endoflare groups demonstrated two file size increases following flaring, while the ProTaper group displayed one file size increase.

Group 1, which did not undergo preflaring, exhibited the highest divergence (257.3 ± 54.4) and revealed a significant difference (p<0.01) compared to all the groups that underwent flaring. The use of HyFlex CM (group 3) for preflaring resulted in the smallest average difference (124.4 ± 29.6) between the maximum diameter of the canal at the apex and the diameter of the initial file. Endoflare (group 4) had the second-smallest average difference (178.7 ± 46) in this regard. Nevertheless, the average variations of the ProTaper (group 2) and Endoflare (group 4) groups did not show any statistically significant differences.

Previous similar investigations utilized maxillary central incisors [15], maxillary premolars [19], mandibular premolars [20], mandibular molars [10], and maxillary molars [21]. The mesiobuccal canals of maxillary first molars were chosen for this investigation because of their significant variation in shape and size. According to Gani and Visvisian, the mesiobuccal canal of the maxillary first molar exhibits an elliptical contour in the apical area, which presents challenges in terms of shaping, cleaning, and filling [11]. Due to the specific configuration of the canal, it is crucial to choose an instrument of the right size that will provide proper canal preparation, primarily in the apical area.

The current study normalized the degree of curvature of the mesiobuccal root of maxillary molars as 10⁰ to 20⁰, applying Schneider's approach [17]. This was accomplished to enhance the dependability of the study results, as the curvature of the root might be seen as an extraneous factor that affects the difference between the dimensions of the canal and the IAF. The process of normalization was examined in prior investigations undertaken by Vanni et al. [21] and Sharma et al. [18]. Since the apical constriction denotes the boundary between periodontal and pulp tissue, it is the optimal location to terminate root canal instrumentation and obturation [22,23]. It was recommended that the canal preparation stop 1 mm before reaching the radiographic apex and apical constriction, which is the designated endpoint for the canal preparation. Additionally, it is recommended to position an apical stop around 0.5 mm to 1 mm away from the apex [24]. Under these recommendations, the total length of the canal in the present investigation was ascertained by introducing a K-file into the canal until the tip of the file appeared apparent at the apex. The WL was subsequently determined at 1 mm less than this measurement [22]. The majority of in vitro research [2,18,25] employed a comparable approach for determining the WL.

Performing the initial estimation of the apical file size without flaring is an erroneous practice, as it can result in a much greater file size after flaring. The study findings demonstrate that the IAF size increased considerably following the flare procedure. The findings were consistent with the earlier research investigated by Pecora et al. [15], Vanni et al. [21], and Sharma et al. [18]. In this investigation, three distinct rotary instruments were used to flare the canal. Following this, hand-held files were put into the mesiobuccal canal of the maxillary molar, beginning with a K-file ISO 08/0.02 at the WL. Hence, the file size increased until the WL was reached and the binding sensation was felt. Following the estimation of the apical file size, the binding instruments were securely placed into the canals at the WL using methacrylate. This approach to determining the initial file size was consistent with research undertaken by Pecora et al. [15], Schmitz et al. [24], Tennert et al. [10], and Sharma et al. [18]. In the majority of the investigations [10,26], the teeth were cut horizontally 1 mm away from the tip, which could potentially impact the binding of the instrument at the WL. In this investigation, the teeth were cut horizontally 0.5 mm away from the tip with a diamond sectioning disk (0.2 mm thick, 1-inch diameter) to reveal the canal and the instrument at the WL. This method safeguards the positioning of the binding instrument (K-file).

Prior research utilized a stereomicroscope [18] and scanning electron microscopy [19,21] to investigate the apical area, enabling visualization of the image from a 2D perspective. The present investigation employed a non-contact 3D optical profilometer to observe the apical slices, which visually scans the entire surface and provides precise measurements in microns and submicrons. Additionally, it offers a substantial working distance. Digital pictures were captured for each sample.

Stabholtz et al. found that increasing the diameter of the coronal part of the canal enhances the operator's potential to accurately locate the apical constriction [27]. This is because the greatest resistance encountered when inserting the file occurs in the coronal one-third of the canal. By initiating a flare at an early stage, the practitioner may accurately assess the size of the canal adjacent to the apex. This enables them to make better choices about the optimal diameter required for apical shaping and cleaning. Our study found that flaring the canal greatly improved the precision of measuring the IAF diameter as compared to root canals that were not flared. Prior research demonstrated greater accuracy in determining the IAF size when early flaring is conducted [28,29]. Tan and Messer [28] observed a single initial rise in apical file size during early flaring, whereas Contreas et al. [29] documented a two-fold increase in IAF sizes following flare. The IAF size before flaring ranged from #10k to #20k, whereas after flaring it ranged from #20k to #30k. The present study results indicate that it is essential to widen the mesiobuccal canal of the maxillary first molar to a greater extent than what was previously considered acceptable. Eliminating coronal interferences facilitates the advancement of the file near the apex, leading to an enlargement in file size. Research on dental anatomy has demonstrated that the size of the apical region of mesiobuccal canals in maxillary molars is equivalent to that of a #25 or #30 file.

The utilization of the #25 file as an MAF for instrumentation in root canal cleaning is not effective, as it aligns with the initial apical binding file size in the mesiobuccal canals of the maxillary first molar, as observed in our investigation. The study found that most of the mesiobuccal canal in maxillary molars had an oval shape, and the researchers measured the canal's greatest diameter. The mesiobuccal canals in the present research were elongated and oval-shaped, with apical diameters that were twice as large as the oval-shaped canals employed in this investigation. To enhance the standardization of the current study, the decision was made to remove elongated oval canals due to their interference with the difference in diameter between the canal and the IAF. The rotary instruments utilized in this investigation, namely ProTaper, HyFlex CM, and Endoflare, exhibit differences in terms of their ISO size and taper. Every system possesses distinct design attributes, resulting in a specific preparation method. The instruments were utilized following the guidelines indicated for each respective system.

The non-flared group exhibited the highest disparity (257.3 ± 54.4) in the present investigation and showed a significant difference compared to all flared groups (p<0.01). The statistical examination of the results revealed notable disparities among the various instrumental groupings. Group 3, which had previously undergone enlargement with HyFlex CM, had the smallest average difference (124.4 ± 29.6) between the IAF diameter and the greatest diameter of the canal at the WL. Group 4 had previously undergone an enlargement procedure using Endoflare and showed the second lowest mean differences (178.7 ± 46) between the diameter of the IAF and the greatest diameter of the canal. The ProTaper group had somewhat higher mean discrepancies (211 ± 43.5). There were statistically insignificant differences in the average values between the ProTaper and HyFlex CM groups.

The minimal disparity in HyFlex CM can be attributed to its distinctive attributes. These files are produced using a distinctive procedure that regulates the material's memory, resulting in files that are very flexible but lack the form memory found in other NiTi files [30]. This provides the file with the capability to precisely adhere to the anatomical structure of the canal. The distinctive characteristic of HyFlex CM files enhances its efficacy in efficiently and expeditiously eliminating dentin projections from the cervical and middle portions of canals. Despite having a significant taper of 12/25, Endoflare's flexibility is limited, resulting in the extent of penetration upon flaring being governed by the resistance experienced at the root canal. The increased disparity in diameter between the IAF and the canal in group 2 (ProTaper group) and group 4 (Endoflare group) can be attributed to their limited flexibility in conforming to the anatomical structure of the canal.

The disparity between the size of the IAF and that of the canal was examined using a destructive approach. This sectioning technique introduces inaccuracies in image interpretation by generating debris surrounding the device. Therefore, the utilization of non-invasive techniques that provide 3D pictures, such as high-resolution micro-computed tomography (micro-CT), could provide significant, precise data in this particular situation. Hence, we recommend that forthcoming inquiries employing a non-invasive imaging technique such as micro-CT yield accurate data concerning the IAF size at different points along the root canal.

## Conclusions

Based on the constraints of this investigation, it can be inferred that coronal preflaring significantly contributes to minimizing the difference between the initial diameter of the apical file and that of the apical canal. The choice of equipment utilized for flaring hinders the accurate estimation of the IAF size. Out of all the groups that flared, the one without preflaring (group 1) had the biggest discrepancy and was substantially different. The use of HyFlex CM (group 3) for preflaring resulted in the smallest average difference between the largest diameter of the canal at the apex and that of the initial file. Endoflare (group 4) had the second-smallest average difference in this regard. Nevertheless, there was a statistically insignificant disparity observed between the average variations in the ProTaper (group 2) and Endoflare (group 4) groups. Consequently, the group that demonstrated the most effective adaptation of the file to the walls of the root canal across all the canals was the group that utilized HyFlex CM rotary NiTi files for enlargement.
